# Cholinergic Markers and Cytokines in OSA Patients

**DOI:** 10.3390/ijms21093264

**Published:** 2020-05-05

**Authors:** Marcella Reale, Lucia Velluto, Marta Di Nicola, Chiara D’Angelo, Erica Costantini, Michele Marchioni, Gianluigi Cerroni, Biancamaria Guarnieri

**Affiliations:** 1 Department of Medical, Oral and Biotechnological Science, University “G. d’Annunzio” Chieti- Pescara, 66100 Chieti, Italy; marta.dinicola@unich.it (M.D.N.); chiara.dangelo@unich.it (C.D.); Erica.costantini@unich.it (E.C.); michele.marchioni@unich.it (M.M.); 2 Center of Sleep Medicine, Department of Neurology, Villa Serena Hospital, Città S. Angelo, 65013 Pescara, Italy; l.velluto@unich.it (L.V.); cerroni_psi@libero.it (G.C.); bmariaguarnieri@villaserena.it (B.G.); 3 Villa Serena Foundation for the Research, Città S. Angelo, 65013 Pescara, Italy

**Keywords:** cholinergic system, cytokines, inflammation, obstructive sleep apnea, polysomnography, sleepiness

## Abstract

The role of inflammation and dysfunction of the cholinergic system in obstructive sleep apnea (OSA) has not exhaustively clarified. Thus, in this study, we explore the non-neuronal cholinergic system and the balance of T helper (Th) 17- and T regulatory (Treg)-related cytokines in OSA patients. The study includes 33 subjects with obstructive sleep apnea and 10 healthy controls (HC). The expression levels of cholinergic system component, RAR-related orphan receptor (RORc), transcription factor forkhead box protein 3 (Foxp3) and cytokines were evaluated. Th17- and Treg-related cytokines, choline levels and acetylcholinesterase (AChE), butyrylcholinesterase (BuChE) activity were quantified in OSA and control subjects. AChE and nicotinic receptor α 7 subunit (α7nAChR) gene expression and serum levels of choline, AChE and BuChE were lower in OSA patients than in the HC group. Compared with the HC group, OSA patients exhibited an increased expression, secretion and serum levels of pro-inflammatory cytokines, a reduced expression, secretion and serum levels of transforming growth factor (TGF)β and reduced Foxp3 mRNA levels. The Th17/Treg-related cytokine ratio was higher in the OSA group. Our results confirm and reinforce the hypothesis that OSA may be considered a systemic inflammatory disease, and that an imbalance of non-neuronal cholinergic and pro/anti-inflammatory cytokines may contribute to development and progression of comorbidities in OSA subjects. The evaluation of Th17/Treg-related cytokine may provide an additional explanation for OSA pathogenesis and clinical features, opening new directions for the OSA management.

## 1. Introduction

OSA is a major primary sleep disorder characterized by repetitive nocturnal complete (apnea) or partial (hypopnea), collapses of the upper airway during sleep accompanied by oxyhemoglobin desaturation and/or electroencephalography (EEG) and vegetative arousals. Nocturnal OSA symptoms include sleep fragmentation, diminished amounts of slow wave and rapid eye movement (REM) sleep, nocturia and insomnia. Daytime symptoms include headache, depressive symptoms, cognitive alterations, fatigue and, in particular excessive daytime sleepiness (EDS), with different patterns between men and women [1,2,3]. EDS is not always present in OSA patients, and it does not invariably correlate with the severity of the disease, showing inter-individual differences with different responses to ventilatory treatments, albeit these aspects need to be better elucidated [4].

The hypothesis that systemic inflammation contributes to OSA risk and to OSA syndrome (OSAS)-associated morbidities is particularly interesting and has been under investigation as far back 1990, though in a small number of patients and with an under-representation of the female population [5,6]. Clinical studies of OSA and inflammation have yielded conflicting results and suggested that there are patient-specific or OSA-specific factors that predict inflammation, but which have not yet been fully identified.

Dyugovskaya et al. showed that in patients with OSA, various subpopulations of T cells acquire an activated phenotype with the downstream consequence of increased cytotoxicity against endothelial cells [7]. Furthermore, this activation process is associated with an increased intracellular content of the pro-inflammatory mediators Tumor necrosis factor (TNF) α and interleukin (IL)-8 and, a decrease of the anti- inflammatory cytokine IL-10 [8], in accord with the observation that in OSA patients repetitive airway obstructions causing intermittent hypoxia induces activation of nuclear factor-κB (NF-kB) and upregulation of pro-inflammatory genes [9].

Over the years many studies have clarified and well documented the bidirectional relationship between the nervous system and the immune system [10] and the expression of most cholinergic system components in immune cells. IL-6 can go through the blood–brain barrier inducing neuroinflammation or neurodegeneration and also causing impairment of neurocognitive functions. The cross talk between the vagus nerve and the immune system was mainly ascribed to inhibiting the immune system [11]. Vagus nerve regulates the production of acetylcholine (ACh) in immune cells, that control inflammation through negative feedback by the binding to α7nAChR expressed by immune cells [11]. Sukys-Claudino et al. have speculated that a deficient cholinergic transmission plays a role in the pathogenesis of OSA in non-Alzheimer’s disease (AD) patients [12] and, a significant role of ACh in wakefulness and breathing was evidenced by Otuyama et al. in individuals with sleep-disordered breathing [13]. The study by Nardone et al. linked, in OSA patients, the impaired cognitive performance with a dysfunction of the cholinergic system [14]. Based on this, our aim is to investigate, in OSA patients, the non-neuronal cholinergic system and the Th17- and Treg-related cytokines and if nicotinic receptor α7 subunit is involved in Th17/Treg polarization and the synthesis of proinflammatory cytokines.

## 2. Results

### 2.1. Participant Characteristics

Our enrolled patients were diagnosed with moderate or severe OSA, 76% have apnea-hypopnea index (AHI) ≥ 30 events per hour and 24% have AHI < 30 events per hour and 54.5% were overweight and 45.5% were obese. No statistically significant difference of selected parameters was found respect to HC group. The demographic characteristics are not different between gender in our OSA patients, the women were all in post-menopausal age (i.e., ≥ 54 years) (Table 1).

Polysomnography (PSG) characteristics and Epworth Sleepiness Scale (ESS) scores of OSA patients enrolled in the study are summarized in Table 2. In OSA patients a significantly and positive correlation between AHI and oxygen desaturation index (ODI) (rho = 0.904, *p* < 0.001) was found. Analysis of polysomnographic indices showed that, in women, wake after sleep onset (WASO) was significantly lower than in men (*p* = 0.042).

### 2.2. Cytokine and Cholinergic Marker Levels

In our OSA subjects we have analyzed serum levels of IL-6 and TGFβ and a significant increase of IL-6 levels (*p* = 0.025) and reduction of TGFβ (*p* = 0.011) was detected compared with HC. To evaluate whether the pattern of serum findings could be related to production by Peripheral Blood Mononuclear Cells (PBMC), we evaluated the spontaneous production of cytokines from PBMC isolated from OSA patients and healthy controls. In cell-free supernatants we detected the lower TGFβ (*p* = 0.048) and higher IL-6 levels, while IL-17 production was not significantly lower in OSA group than in control.

In our OSA patients, we have observed that IL-6 production was inversely correlates with Foxp3 expression (rho = −0.777, *p* = 0.009) and directly with TGFβ production (rho = 0.661, *p* = 0.038).

We have analyzed the relationship between Th17- and Treg-related cytokine evaluating the IL-17/TGFβ ratio in both serum and in the cell-free supernatants. Higher % of IL-17/TGFβ ratio was observed both in serum and cell-free supernatants of patients with OSAS (0.17 and 0.87, respectively) respect to control subjects (0.11 and 0.64, respectively), as summarized in Table 3.

Statistically significant differences of IL-6 and TGFβ serum levels were detected between females and males, as reported in Table 3.

A significantly reduction of choline level and BuChE activity was detected (*p* = 0.004 and *p* = 0.03) in serum of OSA subjects. While it should be observed that no statistically significant differences were observed with respect to gender (Table 3).

### 2.3. Cytokine and Cholinergic Markers Gene Expression

In order to evaluate whether OSAS may influence cytokine expression at transcriptional levels, we analyzed the spontaneous expression of cytokine in PBMC isolated from OSA patients and HC subjects. A significant increase of IL-6 (*p* = 0.029) and IL-17 (*p* = 0.001) gene levels was observed in OSA subjects. A reduction of TGFβ mRNA expression (*p* = 0.214), when compared with control, was detected in OSA patients, confirming the pro-inflammatory environment observed in the serum of OSA patients. Gene expression of transcription factors RORc and Foxp3 was performed; results showed comparable levels in OSA group and in controls. A lower AChE expression was detected in OSA patients, and a statistically significant reduction of α7nAChR mRNA expression was observed in OSA patients when compared with control subjects (*p* = 0.015) (Table 4).

We have analyzed also female and male OSA patient gene expression levels, showing a significantly reduction of Foxp3 (*p* = 0.009) and increased levels of IL-17 in male.

### 2.4. Correlations between PSG Parameters, Gene Expression and Serum Levels of Inflammatory Markers in OSA Patients

**Figure 1:**
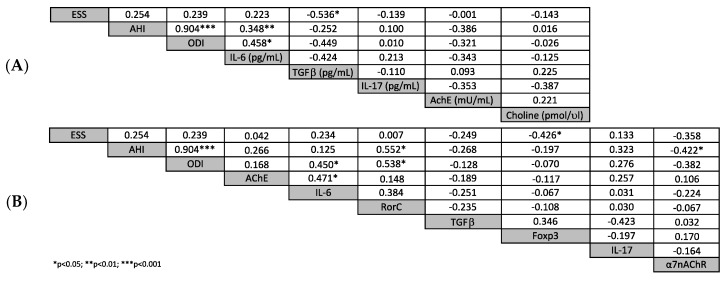
(**A**) Correlation matrix cytokine serum levels and clinical parameters; (**B**) correlation matrix gene expression and clinical parameters.

In OSA patients, a significant negative correlation between ESS scores and serum levels of TGFβ (rho = −0.536; *p* = 0.027) was observed. Serum levels of IL-6 are significantly and positively correlated to ODI (rho = 0.458, *p* = 0.049) and AHI (rho = 0.348, *p* = 0.008) (Figure 1A). RORc gene-expression was significantly correlated with AHI (rho = 0.552; *p* = 0.017) and ODI (rho = 0.538; *p* = 0.023), while a negative correlation was detected between AHI and α7nAChR (rho = −0.422; *p* = 0.042). Interesting, statistically significant correlation between ODI and IL-6 (rho = 0.450, *p* = 0.048) and between Foxp3 gene-expression and sleep functions (rho = −0.426, *p* = 0.049) were detected. (Figure 1B). The correlation analysis was conducted to elucidate if, in our OSA patients, there is a relationship between BMI and inflammatory pathways. Neither the cytokines levels present in the serum, nor the levels present in the supernatant, nor gene expression correlated with the BMI of overall OSA patients, whether female or male OSA.

## 3. Discussion

Sleep and the immune system are closely related, and many functions of the immune system are dependent on circadian rhythms and regular sleep. Sleep disorders can cause changes in the function of immune cells, particularly T cells, increases of inflammatory mediators and, on the other hand, inflammatory mediators that are released during immune responses alter the central nervous system (CNS) processes and behavior, including sleep. In the last two decades many studies have highlighted a solid link between OSA and systemic inflammation and have sought to clarify the relationships between OSA and inflammatory pathways. Consequence of hypoxia/reoxygenation phenomena, a characteristic aspect of OSA, is the activation of NF-kB and increased production of inflammatory cytokines [15] and the differentiation of Th17 cells [16] that are characterized by the expression of retinoic acid-receptor (RAR)-related orphan receptor (ROR)γt transcription factor, encoded by gene RORc [17] and by the production of IL-17, a cytokine that play a critical role in inflammatory, autoimmune and infectious diseases. T cells, expressing Foxp and named Treg cells, orchestrate the overall immune response, regulating the activity of the effector T cells or releasing anti-inflammatory cytokines. The balance between Foxp3+ Treg and Th17 cells was determined by IL-6 that modulates the TGFβ-induced generation of Foxp3+ Treg and drive Th17 cell differentiation. The balance between TGFβ, an immunosuppressive cytokine, and IL-6—a pro-inflammatory cytokine—may influence the final outcome in the differentiation process of different effector T cell subsets [18].

In this study, we observed significantly higher IL-6 and IL-17 and gene expression in PBMC of OSA patients and significantly higher IL-6 and lower TGFβ serum and cell-free levels compared to HC, in accord to the established modulatory effect of IL-6 and TGFβ on generation of Foxp3+ cells [19].

Our experiments suggested that in the micro-environment of OSA patients, the increase of pro- inflammatory IL-6 and the reduction of TGFβ potentially promote the Th17/Treg imbalance. The shift of IL-17/TGFβ balance toward IL-17 may enhance the accumulation of inflammatory mediators, and finally generate a proinflammatory loop to amplify proinflammatory environment that in moderate-severe OSA patients shift the balance between Th1/Treg cells toward Th1 cells.

Although IL-17 has no significant higher circulating levels in our OSA patients, taking into account its significantly high expression levels, we cannot exclude a role for IL-17 in OSA-related inflammation. Thus, IL-17 in conjunction with reduced TGFβ, modulate the development of Treg cells, causing a Th17/Treg imbalance. Our results are in accordance with the study of Ye et al. [18] that suggested that an elevated Th17/Treg ratio may be a marker of inflammatory and autoimmune diseases and supported the hypothesis that OSAS induces a systemic inflammatory response activating the signal transduction pathway leading to upregulation of inflammatory cytokines and downregulation of anti-inflammatory once.

**Figure 2:**
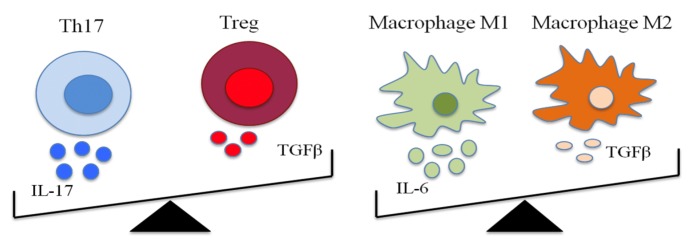
Schematic representation of suggested dynamic balance between pro-inflammatory and anti-inflammatory cytokines in OSA.

An intriguing hypothesis that could involve the balance between Th1 cells and Treg cells as well as balance between M1 and M2 that could be shifted, respectively by up- regulation of IL-6 and down-regulation of TGFβ toward an inflammatory Th1 and M1 cell phenotype, respectively (Figure 2). Further investigations will be needed to understand the role of proinflammatory and anti-inflammatory balance in the activation of different downstream pathophysiological cascades, in OSA patients.

In accord with the study by Freire et al, showing that OSA-induced sleep interruption is not associated with lymphocyte, neutrophil or peripheral blood cell count alterations [20], in our OSA patients no statistically significant association was found between AHI or BMI and white blood cells (WBCs), lymphocytes, neutrophils and total lymphocyte count, confirming that the different serum levels of cytokines are related to cells polarization and not to their number.

To elucidate if, in our OSA patients, there is a relationship between BMI and inflammatory pathways the correlation analysis was conduct. Neither the cytokines levels in the serum or in the supernatant, nor their gene expression correlates with the BMI of overall OSA patients, whether female or male.

Previous studies have suggested that peripheral pro-inflammatory cytokines such as IL-1 and IL-6 can go through the blood–brain barrier and activate and regulate the vagus nerve system, affecting the function of central nervous system. The cross-talk between peripheral immune system and the CNS has well characterized and is the vagus nerve-based “inflammatory reflex” that regulates inflammation and immune responses [10]. The role of cholinergic system in wakefulness and cognition, in attentional, memory and coordination processes [21] in neurodegenerative disease was widely studied and the relationship between brain cholinergic dysfunction, neuroinflammation and peripheral immune dysregulation was highlighted [22,23].

We have explored a non-neuronal cholinergic system in subjects with and without OSA in order to clarify the potential link between inflammation, sleepiness and non-neuronal cholinergic system.

The cholinergic system may be involved in the pathophysiology of respiratory disorders, such as sleep apnea, affecting the regulation of breathing during sleep and in the activation of brain areas involved in arousal and vigilance levels.

The pilot study of Pak et al. showed that lower levels of plasma choline metabolites are associated with sleepiness [24], but whether low choline is a result of disturbed sleep with subsequent sleepiness was not determined. In our patients we detected a significantly reduction of choline and BuChE activity and α7nAChR expression pointing out that non-neuronal cholinergic system may be associated with OSA. Our results are in accord with the study of Xue et al. that in mice subjected to sleep disturbances detected a down-regulation of α7nAChR and an increase of pro-inflammatory cytokines in microglia and astrocytes [25]. In our OSA patients we detected a weak or absent correlation between α7nAChR expression and inflammatory cytokines. Thus, further studies are needed to confirm, in OSA patients, the link between α7nAChR expression, inflammatory status and modulation of neurocognitive, cardiovascular, immune or metabolic functions and to understand if sleep disordered breathing modulate the cholinergic system and inflammation, or vice versa.

Even if the underlying mechanisms leading to OSA-induced morbidities are probably multifactorial and are not fully clarified, it is evident that activation of the inflammatory pathways is an important pathophysiological component of cardiovascular, metabolic, behavioral and neurocognitive morbidities. OSA is associated with increased risk of developing cardiovascular, immune functions behavioral and cognitive dysfunction that may be silent, progressive and potentially reversible during earlier stages; thus, an early assessment of the inflammatory state can help the management of disease.

Recently, more attention has been given to the evaluation of differences in OSA clinical and PSG profiles in men and women [26,27]. Menopause is a risk factor for sleep-disordered breathing in women, but in the aging men and women a comparable incidence of OSA was detected [28]. While the influence of alcohol consumption and smoking habit has been associated with a higher risk and frequency of OSA in men. In our male and female OSA patients, sleepiness status was not significantly different, although lower score of disease severity was observed in the female group, as well as serum cytokine levels. Further studies addressing gender differences in patients with OSA are needed to clarify if pathogenic mechanisms are different in men and women and to lead the discovery of new biomarkers, as well as new targets for personalized treatment.

## 4. Materials and Methods

### 4.1. Subjects

Thirty-three patients—12 female and 21 male, with median age of 30.3 years (IQR: 27.3–33.5), with Polysomnography confirmed moderate-severe OSA diagnosis—were investigated. Ten healthy controls, frequency matched for age and gender with OSA group and without any chronic inflammatory/autoimmune diseases and did not receive any treatments were enrolled. Berlin questionnaire and a deep medical/sleep interview were used in HC to exclude OSA syndrome. No subjects with other significant primary sleep disturbances were included. All participants completed medical and sleep history with a physician and a psychologist both expert in sleep medicine. Moreover, they filled self-administered scales to assess sleep and excessive daytime sleepiness (EDS) [29]. EDS was diagnosed when the ESS score exceeded 10. The international restless legs syndrome (RLS) study group criteria for the clinical diagnosis of RLS was used [30]. The Berlin questionnaire was used for OSA risk assessment [31]. The study was approved by the Ethics Committee of Chieti-Pescara (n 14; 01.09.2016; sic. richef3md) and all the participants gave informed consent. All patients underwent a full-night video PSG at the Center of Sleep Medicine, department of Neurology, Villa Serena Hospital performed according to the American Academy of sleep medicine [32]. The OSA diagnosis and degree (moderate or severe) were established in accordance with the AASM manual for the scoring of sleep and associated events and the International Classification Of Sleep Disorders (ICSD) third edition 2014. PSG parameters evaluated are: apnea-hypopnea index (AHI); excessive daytime sleepiness (EDS); oxygen desaturation index (ODI); total sleep time (TST); Epworth sleepiness scale (ESS); time of oxygen saturation levels < 90% (T90); wake after sleep onset (WASO). In our population, 76% had AHI ≥ 30 events per hour and 24% had AHI < 30 events per hour.

### 4.2. Demographic and Laboratory Data

The demographic and clinical data were obtained from all the participants, including age, weight, body mass index (BMI), history of smoking status. The history of hypertension, stroke and diabetes were considered although all subjects were in good clinical compensation with ongoing therapy. In each patient fasting complete VES and blood count were analyzed.

### 4.3. Serum Samples Collection and PBMC Purification

Peripheral venous blood samples (3–5 mL) were collected in the morning between 08:00 and 10:00 in vacutainer tubes containing sodium heparin, according to the routine puncture method. Serum was collected by blood centrifugation at 1600 rpm for 10 min and frozen at −80 °C within 30 min, until assayed. Ten milliliters of blood/saline (1:2 *v*/*v*) was layered over 5 mL of Ficoll-Paque (Sigma-Aldrich, Italy) and centrifuged at 1600 rpm for 30 min at room temperature. Cells were harvested from the interface, washed twice with Phosphate Buffer Saline (PBS) and resuspended in RPMI1640 medium (Sigma-Aldrich, Italy) supplemented with 10% fetal bovine serum (FBS) (EuroClone, MI, Italy), 4-mM L-glutamine, 50 U/mL penicillin and 50 mg/L streptomycin (all purchased by Sigma-Aldrich, Italy). PBMC (3 × 106 in 1 mL of medium) were placed in 5 mL polypropylene culture tubes (BD Falcon™, Two Oak Park Bedford, MA, USA) and incubated at 37 °C in 5% CO_2_ in cell culture incubator for 24 h. After this time, tubes were centrifuged to collect separately cell pellet and supernatant that were stored at −80 °C for later analysis of spontaneous cytokines expression and production. Biosafety level 2 procedures were applied for working with patients’ samples.

### 4.4. Cytokine Measurements

Cytokines levels were measured using specific Human ELISA kit (Diaclone, Besancon Cedex, France) according to the manufacturer’s instructions. The plates were read at λ 450 nm (GloMax^®^ Multi Detection System, Promega, MI, Italy) and the absorbance was transformed to pg/mL, using calibration curves prepared with cytokine standards included in the kits. The intra- and inter-assay reproducibility were > 90%. The specificity and the sensitivity for the cytokines were defined according to the manufacturer. Minimum detectable dose (MDD) was 2 pg/mL for IL-6 and assay range between 0 and 200 pg/mL; < 2.3 pg/mL for IL-17A with a range assay between 0 and 100 pg/mL and MDD of 8.6 pg/mL for TGFβ with assay range between 0 and 2000 pg/mL.

### 4.5. Measurement of Serum Choline, AChE and BuChE Activity

Total choline (free choline + ACh) concentration and AChE and BuChE activities were measured by commercial colorimetric kit (BioVision, CA, USA). The level of choline/ACh (pmol/µL) was calculated by plotting the absorbance of each sample in relation to choline standard curve. The assay rage was from 0 to 500 pmol/µL. Hydrolyzing activity of both cholinesterases were reported as mU/mL. The measurement was obtained using GloMax^®^ Multi Detection System (Promega, MI, Italy) at λ 570 nm.

### 4.6. RNA Extraction, RT and Real-Time PCR

Total RNA was extracted from PBMCs using QIAzol reagent (Qiagen, Hilden, Germany) according to the manufacturer’s protocol. The RNA concentration was determined by measuring the samples’ absorbance at 260 nm by NanoDrop 2000 UV-Vis Spectrophotometer (Thermo Scientific, Waltham, MA, USA) and its purity was assessed by the absorbance ratio 260/280 nm and 260/230 nm. For each sample, 1 µg of RNA was reverse transcribed into complementary DNA (cDNA) using QuantiTect Reverse Transcription Kit (Qiagen, Hilden, Germany). Subsequently, Real-Time PCR was performed using the GoTaq^®^ qPCR Master Mix (Promega, MI, Italy), to evaluate the gene expression of IL-6, IL-17, TGFβ, Foxp3, RORc, AChE, α7nAChR and RPS18, used as reference gene. All PCR reactions were performed in triplicates using the Mastercycler ep (Eppendorf, Hamburg, Germany) with the following conditions: initially, 2-min incubation at 95 °C followed by 40 cycles consisting of 30 s at 95 °C, then 60 °C for 1 min and 30 s at 68 °C. The gene expression analysis was done according to ΔΔ*C*t method [33].

### 4.7. Statistical Analysis

Quantitative variables were summarized as mean ± standard deviation (SD) or as median and interquartile range (IQR) according to their distribution evaluated by Shapiro–Wilk test. Categorical variables were reported as frequencies and percentages.

Patients were stratified according to the OSA status and differences between the two conditions were tested by the non-parametric Mann–Whitney *U* test and Pearson chi-squared test for continuous and categorical variables, respectively. Spearman Rho correlation coefficients (Rho) were computed to evaluate the correlation between serum cytokine levels, gene expression and clinical parameters within the OSA group. Correlations were graphically depicted as correlation matrixes.

To give an indication of the precision of the fold change, calculated with 2^−ΔΔ*C*t^ method, the 95% confidence interval (95% CI) was determined. Student t-test for one sample was applied to evaluate statistical differences in mRNA gene expression levels between OSA patients and their matched HC with predetermined value equal to 1. Student *t*-test for unpaired data were applied to evaluate the differences in mRNA gene expression levels between male and female patients. All tests were 2-sided and a level of statistical significance was set at *p* < 0.05. Analyses were performed using the R software environment for statistical computing and graphics (version 3.5.3; http://www.r-project.org/).

## 5. Conclusions

This preliminary study has some limitations. First of all, it may be biased, since the small differences in subject characteristics and the sample size, does not allow to significantly categorize male and female group and establish a clear relationship between sex and cholinergic and immune system in OSA patients. Since only 24% of patients have 15 AHI < 30, evaluation of cholinergic and immunological parameters did not give significantly differences respect to patients with AHI > 30.

Although underpowered, this study may provide interesting results for further investigation to better asses the mechanistic and functional relationship between sleep disordered breathing, inflammatory cytokines and non-neuronal cholinergic systems in males and females.

Here we hypothesize that in OSA patients the impairment of α7nAChR expression contributes to exacerbate inflammatory conditions making these patients more susceptible to comorbidity development. To the best of our knowledge, this is the first study to show the outcome of cholinergic and inflammatory system modulation in OSA patients. The complete mechanism through which OSA may be linked to impaired cholinergic pathway and if it could influence immune functions and metabolic or cardiovascular disease risk is not completely clarified. Thus, the results of this study can pave the way for new studies on the role of both inflammatory and cholinergic markers in OSA prognosis, comorbidities and related treatments in men and women.

## Figures and Tables

**Table 1:** Demographic and laboratory data of obstructive sleep apnea (OSA) and healthy controls (HC).

Variable	OSA	HC	Mann–Whitney *U* Test OSA vs. HC	OSA Females	OSA Males	Mann–Whitney *U* Test OSA Females vs. OSA Males
Gender (M/F), *n*	21/12	5/5	0.687 ^a^			
Age (years), median (IQR)	63.0 (54.0–69.0)	57.0 (54.5–60.0)	0.187	66.5 (62.2–69.0)	60.0 (53.0–65.0)	0.132
BMI (kg/m^2^), median (IQR)	30.3 (27.3–33.5)	25.0 (24.1–26.0)	0.002	31.0 (29.6–34.9)	28.7 (26.3–33.4)	0.186
Smoking habits (Yes), *n* (%)	4 (12.1)	2 (20.0)	0.913 ^a^	2 (16.7)	3 (14.3)	0.854 ^a^
Diabetes, *n* (%)	6 (18.2)	1 (10.0)	0.900 ^a^	2 (16.7)	4 (19.0)	0.864 ^a^
Hypertension, *n* (%)	22 (66.7)	5 (50.0)	0.561 ^a^	8 (66.7)	13 (61.9)	0.784 ^a^
VES (mm/h), median (IQR)	9.0 (6.0–13.5)	12.0 (8.0–14.4)	0.169	11.5 (9.2–15.5)	6.0 (5.0–12.7)	0.090
WBC (10^3^/µL), median (IQR)	7.3 (6.2–8.6)	6.8 (5.0–7.9)	0.294	7.5 (6.9–8.7)	6.7 (6.1–8.8)	0.706
Lymphocytes %, median (IQR)	32.2 (24.0–35.4)	31.5 (23.5–34.2)	0.572	32.9 (30.8–37.3)	28.5 (23.6–34.1)	0.078
Monocytes %, median (IQR)	8.1 (7.2–9.4)	7.5 (6.5–10.0)	0.242	7.9 (7.1–9.0)	8.2 (7.2–9.9)	0.488
Neutrophils %, median (IQR)	56.1 (53.0–62.9)	55.4 (52.0–63.5)	0.325	55.8 (51.1–59.1)	56.2 (53.5–65.3)	0.137
RBC (10^6^/µL), median (IQR)	5.0 (4.7–5.3)	5.3 (4.0–6.3)	0.448	4.8 (4.7–5.1)	5.2 (4.7–5.4)	0.094

^a^*p* value relative to chi-squared test comparison between OSA and HC groups or between OSA females and OSA males. IQR: interquartile range (Q_1_–Q_3_).

**Table 2:** Clinical details of overall OSA patients and scores of female and male enrolled. Data are expressed as median and interquartile range (Q_1_–Q_3_).

Variable	OSA	OSA Females	OSA Males	Mann–Whitney *U* Test OSA Females vs. OSA Males
ESS score	11 (6–14)	10.5 (5–12)	11.0 (6.0–14.5)	0.762
TST (min)	424.4 (383.5–470.0)	426.2 (384.2–444.7)	424.4 (382.0–477.7)	0.577
AHI (episodes/h)	36.6 (27.4–61.2)	35.4 (33.4–71.2)	38.4 (27.4–55.9)	0.779
T90 (%)	15.7 (4.9–40.0)	15.7 (7.5–29.7)	16.3 (2.1–44.5)	0.754
WASO	58.5 (37.2–88.3)	50.7 (24.4–59.5)	70.2 (37.3–110.1)	0.042
ODI	35.8 (18.0–56.2)	38.1 (24.3–67.1)	33.0 (16.1–56.2)	0.306
Sleep efficiency%	86.2 (79.1–89.1)	85.6 (80.7–88.9)	86.3 (75.6–89.1)	0.527

**Table 3:** Cytokines and cholinergic marker levels in serum and cell-free supernatant of OSA and HC groups. Data are expressed as median and interquartile range (Q1–Q3).

Serum	OSA	HC	Mann–Whitney *U* Test of HC vs. OSA	OSA Female	OSA Male	Mann–Whitney *U* Test OSA Female vs. OSA Male
IL-6 (pg/mL)	4.9 (4.7–5.5)	4.4 (4.3–5.0)	0.025	5.1 (4.7–5.7)	4.9 (4.7–5.3)	0.008
IL-17 (pg/mL)	2.6 (0.8–3.2)	2.6 (1.7–3.1)	0.792	2.7 (1.0–3.5)	1.8 (0.6–3.8)	0.716
TGFβ (pg/mL)	1405.4 (831.9–1811.5)	2034.3 (1677.9–2391.2)	0.011	1510.7 (909.9–1935.4)	1350.0 (654.6–1659.5)	0.046
IL-17/TGFβ (%)	0.17 (0.09–0.31)	0.11 (0.08–0.20)	0.312	0.17 (0.13–0.28)	0.23 (0.04–0.35)	0.976
Choline (pmol/µL)	0.32 (0.31–0.34)	0.36 (0.33–0.40)	0.004	0.33 (0.31–0.36)	0.31 (0.31–0.33)	0.509
AChE (mU/mL)	6.54 (5.75–6.82)	6.96 (6.14–7.30)	0.438	6.49 (5.72–6.75)	6.61 (5.77–7.18)	0.371
BuChE (mU/mL)	14.46 (10.84–18.93)	17.95 (15.93–24.73)	0.031	13.45 (10.24–17.13)	16.58 (10.68–19.69)	0.843
Cell-free Supernatant						
IL-6 (pg/mL)	283.4 (107.2–227.8)	60.7 (40.7–96.4)	0.042	142.3 (36.3–220.7)	220.8 (147.6–336.2)	0.899
IL-17 (pg/mL)	2.3 (1.5–4.1)	2.8 (2.6–3.1)	0.611	2.0 (1.5–4.5)	3.1 (1.8–4.3)	0.904
TGFβ (pg/mL)	183.4 (142.3–278.2)	330.9 (192.4–792.5)	0.048	176.5 (140.2–258.2)	190.2 (146.0–298.7)	0.770
IL-17/TGFβ (%)	0.87 (0.65–1.7)	0.64 (0.36–1.6)	0.283	0.83 (0.67–1.08)	1.53 (0.59–1.81)	0.660

**Table 4:** Cytokine and cholinergic markers gene expression (2^−ΔΔ*C*t^) in OSA group. Data are expressed as mean and 95% confidence interval (95% CI).

Variable	OSA	Student t-Test OSA vs. HC	OSA Female	OSA Male	Student *t*-Test OSA Female vs. OSA Male
IL-6	2.52 (1.23–5.16)	0.029	2.77 (0.59–12.96)	3.15 (0.60–16.44)	0.382
IL-17	6.51 (1.31–32.24)	0.001	3.32 (0.08–126.70)	12.90 (0.83–198.92)	0.281
TGFβ	0.77 (0.37–1.60)	0.214	0.72 (0.23–2.22)	0.75 (0.29–2.07)	0.431
Foxp3	0.90 (0.25–3.21)	0.669	3.20 (0.52–19.73)	0.47 (0.07–3.07)	0.009
RORc	0.98 (0.32–2.98)	0.463	1.60 (0.45–5.58)	0.49 (0.08–2.86)	0.991
AChE	0.72 (0.45–1.16)	0.053	0.71 (0.34–1.47)	0.62 (0.34–1.11)	0.833
BuChE	1.08 (0.29–25.78)	0.678	1.07 (0.56–2.01)	1.08 (0.02–49.55)	0.475
α7nAChR	0.60 (0.32–0.92)	0.015	0.65 (0.30–1.44)	0.47 (0.18–1.22)	0.957

## Abbreviations

AHI	Apnea-hypopnea index number of apnea/hypopnea events per hour of sleep
BMI	Body mass index
EDS	Excessive daytime sleepiness
ODI	Oxygen desaturation index. The average number of desaturations of 3% or more per hour of sleep
TST	Percentage of total sleep time spent with an oxyhemoglobin saturation below 90% with SaO2 < 90%
ESS	Epworth sleepiness scale
T90	Percentage of epoch spent at oxygen saturation levels < 90%. T90 ≥ 30% indicates Nocturnal respiratory failure
WASO	wake after sleep onset
